# Neurocognitive Function in Acromegaly after Surgical Resection of GH-Secreting Adenoma versus Naïve Acromegaly

**DOI:** 10.1371/journal.pone.0060041

**Published:** 2013-04-04

**Authors:** Juan Francisco Martín-Rodríguez, Ainara Madrazo-Atutxa, Eva Venegas-Moreno, Pedro Benito-López, María Ángeles Gálvez, David A. Cano, Francisco J. Tinahones, Elena Torres-Vela, Alfonso Soto-Moreno, Alfonso Leal-Cerro

**Affiliations:** 1 Instituto de Biomedicina de Sevilla (IBiS), University Hospital Virgen del Rocío/Consejo Superior de Investigaciones Científicas/University of Seville and Division of Endocrinology, Virgen del Rocío University Hospital, Seville, Spain; 2 Division of Endocrinology, Hospital Universitario Reina Sofía, University of Córdoba, Córdoba, Spain; 3 Division of Endocrinology, Virgen de la Victoria Hospital, Málaga, Spain; 4 Division of Endocrinology, San Cecilio Hospital, Granada, Spain; John Hopkins University School of Medicine, United States of America

## Abstract

Patients with active untreated acromegaly show mild to moderate neurocognitive disorders that are associated to chronic exposure to growth hormone (GH) and insulin-like growth factor (IGF-I) hypersecretion. However, it is unknown whether these disorders improve after controlling GH/IGF-I hypersecretion. The aim of this study was to compare neurocognitive functions of patients who successfully underwent GH-secreting adenoma transsphenoidal surgery (cured patients) with patients with naive acromegaly. In addition, we wanted to determine the impact of different clinical and biochemical variables on neurocognitive status in patients with active disease and after long-term cure. A battery of six standardized neuropsychological tests assessed attention, memory and executive functioning. In addition, a quantitative electroencephalography with Low-Resolution Electromagnetic Tomography (LORETA) solution was performed to obtain information about the neurophysiological state of the patients. Neurocognitive data was compared to that of a healthy control group. Multiple linear regression analysis was also conducted using clinical and hormonal parameters to obtain a set of independent predictors of neurocognitive state before and after cure. Both groups of patients scored significantly poorer than the healthy controls on memory tests, especially those assessing visual and verbal recall. Patients with cured acromegaly did not obtain better cognitive measures than naïve patients. Furthermore memory deficits were associated with decreased beta activity in left medial temporal cortex in both groups of patients. Regression analysis showed longer duration of untreated acromegaly was associated with more severe neurocognitive complications, regardless of the diagnostic group, whereas GH levels at the time of assessment was related to neurocognitive outcome only in naïve patients. Longer duration of post-operative biochemical remission of acromegaly was associated with better neurocognitive state. Overall, this data suggests that the effects of chronic exposure to GH/IGF-I hypersecretion could have long-term effects on brain functions.

## Introduction

Acromegaly is a rare but severe hormonal disorder resulting from aberrant GH (growth hormone) secretion and consequent increase of IGF-I (insulin-like growth factor 1), most commonly produced by pituitary adenomas. The development of this disease is insidious, with a mean duration of 5–10 years from symptom onset to diagnosis. Systemic complications are also common at the time of diagnosis, including changes in the somatic, gastrointestinal, cardiovascular, endocrine and metabolic systems, among others [1], [2].

Neurocognitive complications, including working memory and long-term memory deficits, have been added to the long list of complications associated with this disease [3]–[5]. Increasing evidence suggests that the severity of these complications in acromegaly patients is strongly correlated to circulating GH/IGF-I levels and the duration of active disease, apparently independent from psychiatric symptoms [3], [4], [6]. Recent magnetic resonance imaging (MRI) studies have reported structural alterations in grey and white brain matter (including the hippocampus) in acromegaly patients [5], [7]. Moreover, neurophysiological alterations have been also described in active untreated (naïve) patients. These patients display decreased amplitude of the cognitive potential P300 under the auditory oddball paradigm [3] as well as decreased alpha and beta activity in specific cortical regions such as prefrontal cortex (PFC) and medial temporal cortex (MTC) [4]. Altogether, these observations strongly suggest that prolonged GH/IGF-I excess may have a negative effect on neural and cognitive function.

Nevertheless, few neurocognitive studies to date have been conducted in cured acromegaly patients. The study of cured acromegaly patients (patients in which GH/IGF-I levels have been restored to normal concentrations) provides a unique opportunity to examine the potential reversibility of alterations in brain structure and function induced by GH and IGF-I excess. The first therapeutic approach to active acromegaly is adenoma removal, chiefly through transsphenoidal neurosurgery. However, despite successful control of GH/IGF-I hypersecretion, this approach does not always result in the complete relief of acromegaly-related comorbidity. Severe cardiovascular complications and lesser disorders, such as sleep apnea and incapacitating arthropathy, are commonly observed in cured patients [8]–[10]. These complications contribute significantly to the decreased quality of life and high rate of psychiatric disorders often associated with acromegaly patients [11].

Our previous research had revealed that patients with active untreated acromegaly show specific neurocognitive impairment associated with increased GH and IGF-I levels [4]. In the present study, we investigated the extent of neurocognitive impairment after biochemical remission of acromegaly. To this end, we assessed the neurophysiological and cognitive status of acromegaly patients after surgical cure. To avoid potential confounding effects of current adjuvant treatments for acromegaly (pharmacologically and/or radiotherapy) in the neurocognitive assessment, our analysis was restricted to patients cured by transsphenoidal surgery alone. Based on our previous results, we tested the hypotheses that cured acromegaly patients show better neurocognitive status than patients with active disease, and that the persistence of neurocognitive disorders after cure is linked to the duration of GH and IGF-I hypersecretion. To test these hypotheses, we focused on a set of clinical and biochemical variables that have previously shown to correlate to neurocognitive status in acromegaly [3]–[5], [7]. However, previous work has been restricted uniquely to univariate correlations. In the current work, we performed a more comprehensive analysis from a multivariate analysis approach that includes clinical, demographical and biochemical variables, thus controlling for confounding effects, interactions and multicollinearity between variables.

We decided to focus on memory functions and their relationship to PFC and MTP regions since previous studies have described that these brain regions are functionally altered in active acromegaly [4], [12]. For comparison purposes, a group of patients with naïve (active untreated) acromegaly was included in the study.

## Materials and Methods

### Subjects

We obtained data on 102 patients diagnosed with acromegaly between 1990 and 2010 from the databases of two University hospitals in Andalusia, Spain. In all cases, acromegaly had been diagnosed by the presence of relevant clinical signs: IGF-I above the age-specific normal range (IGF-I_SD_) and failure to reduce GH levels below 1 µg/liter during oral glucose tolerance tests (OGTT). Patients with major psychiatric syndrome or neurological background, history of pituitary radiotherapy, untreated hypopituitarism, uncontrolled diabetes mellitus, or uncontrolled hypertension were considered ineligible. Also excluded were patients that had undergone transcranial surgery for pituitary adenoma removal (Figure S1).

Patients with cured acromegaly were defined based on last published consensus criteria (GH nadir below 0.4 µg/liter after a post-operative 75-g OGTT and normal IGF-I_SD_ level) [13]. The patients included in this study had been cured of acromegaly (according to the criteria described above) the preceding 6 months (at a minimum) before the neurocognitive assessments. A total of thirty patients met this criteria and were invited to participate in a neurophysiological and cognitive assessment. Eight patients declined (four noted incompatibility with work schedules, four gave no explanation). Two patients did not complete the assessment. The demographic and clinical characteristics of these ten patients did not differ from those patients included in the final sample.

Patients with naïve acromegaly were also drawn from the hospital databases. These newly diagnosed patients were on waiting lists for transsphenoidal tumor resection. Exclusion criteria included major psychiatric syndrome or neurological background, uncontrolled diabetes mellitus, uncontrolled hypertension or untreated hypopituitarism. Each patient from this group was matched on gender and educational level to a patient in the cured acromegaly group. Age matching was limited to a five-year age difference. None of these patients had previously performed the tests used in the current study.

Anthropometrical measures and blood sample collection for standard biochemical analysis and hormonal parameters (GH, IGF-I, PRL, LH, FSH, serum T4, TSH, 0900-h cortisol, testosterone or estradiol) were performed under basal conditions, between 8∶00 am and 9∶00 am, after an overnight fast (see Supplementary information for hormonal assay description in File S1).

Twenty demographically matched control healthy subjects were included in the study. Exclusion criteria for this group included any history of endocrine or neurological disease, and present or previous substance abuse. Age did not differ among groups (see Table S1 for demographic features of the groups). Subjects from this sample were selected from a larger sample of voluntary healthy subjects who served as individual controls in an unpublished study on the prevalence of memory complaints in patients with pituitary tumors. None of the control subjects had previously performed the tests used in the current study. The study protocol was approved by the Virgen del Rocio University Hospital Medical Ethics Committee (report number: A08011074). Written informed consent was obtained from all participants.

### Cognitive Measures

To assess cognitive functions, we applied six standardized neuropsychological tests, selected on the basis of proven sensitivity to cognitive deficits in neuroendocrine disease [4], [12]. These tests were classified and subdivided into four cognitive domains: attention, executive functioning, working memory and delayed memory recall (verbal and visual) [14]. The tests included two computerized versions of the Letter Cancellation Tasks, the Tower of Hanoi and the Stroop Color-Word Test, the Trail-Making Test (B), Luria’s Memory Words Test-Revisited (LMW-R), the Complex FigTest (CFT), and the Digit Span Test (see File S1 for more details).

### Neurophysiological Measures

Resting-state brain function was assessed using electroencephalography (EEG) data from a 32-channel digital EEG recorder (linked-ears reference, bandwidth [0.1–100 Hz], sampling frequency = 256 Hz), with electrode placement in accordance with the 10–10 system [15]. Each participant underwent a three-minute resting state EEG recording (eyes closed). Raw data was processed and filtered offline using EEGLAB [16]. Power spectral analysis (1–40 Hz, 1 Hz resolution) was conducted on artifact-free EEG segments using the Fast Fourier Transform (FFT). We then averaged data across frequency band and scalp location to obtain measures for frontal, central-temporal and parietal-occipital electrode placement.

We also applied Low-Resolution Electromagnetic Tomography (LORETA) solutions to locate the source of EEG activity from the scalp-recorded data [17]. LORETA is a functional imaging technique that models EEG sources into 3D distributions [18], providing a linear solution to the inverse EEG problem. This technique uses a three-shell spherical head model that includes scalp, skull and brain compartments. The latter is restricted to the cortical gray matter and hippocampus of a head model co-registered to the Talairach probability atlas, digitized at the Brain Imaging Center of the Montreal Neurological Institute [19]. This compartment includes 2,394 voxels (7 mm resolution), each containing an equivalent current dipole. We obtained LORETA values for the following frequency bands: delta (1–3 Hz), theta (4–7 Hz), alpha (8–12 Hz), beta 1 (13–18 Hz), beta 2 (19–21 Hz), beta 3 (22–30 Hz), and gamma (31–40 Hz). We performed region of interest (ROI) analysis on the PFC (Brodmann areas 8 to 11 and 44 to 47) [20] and the MTC (Brodmann areas 27, 28, 34 to 36, including the fusiform gyrus) [21].

### Quality of Life (QoL) Measure

A self-administered questionnaire was used to assess the impact of acromegaly on patients’ QoL. The Acromegaly Quality of Life Questionnaire (AcroQoL), specifically developed for QoL assessment in patients with acromegaly [22], was used. This questionnaire assesses both the physical and psychological aspects of the disease. AcroQol was not administered to healthy subjects.

### Statistical Analysis

The Fisher exact probability test was used to evaluate the association between categorical variables. For general cognitive and memory tests, one-way and repeated measures ANOVA/ANCOVA tests were chosen, respectively. In these analyses, covariates included number of comorbidities and QoL status. The familywise error rate corrected *p*-value was estimated using 5,000 permutations. A Bonferroni correction was used for pairwise post hoc comparisons.

The EEG power spectral analysis (from 1–40 Hz) was grouped into classic frequency bands. We used non-parametric statistical mapping to analyze the LORETA results. After applying subject-wise normalization, we conducted group comparisons using voxel-by-voxel permutation testing. To assess statistical differences in our ROIs, exact randomization probabilities (5,000 randomizations), corrected for multiple comparisons, were calculated to test cluster size. To study possible relationships between clinical parameters and neurocognitive variables, we carried out group level analyses using a random-effects ordinary least squares model that included the following predictors: 1) constant (overall mean), 2) patient group (cured, naïve, i.e. effects coding 1 or −1), 3) GH levels, 4) GH levels×diagnostic group, 5) duration of untreated disease, 6) duration of untreated disease×diagnostic group. Additional covariates included age at diagnosis, Qol status, prolactin levels, hypertension, diabetes and hypopituitarism. The variable ‘duration of untreated disease’ (the time the patient was exposed to increased IGF-I or GH without treatment) was estimated on each patient as described elsewhere [7]. In addition, the impact of the duration of the biochemical remission (defined as the period between GH/IGF-I normalization and neurocognitive testing in treated patients) on neurocognitive functions was assessed in a linear regression analysis. IGF-I levels were not entered in this analysis to avoid multicollinearity with GH levels. In order to obtain predictive capacity of clinical variables on abnormal results, dependent variables were transformed into Z-scores using data from healthy subjects. Given that residuals of the regression equations were not normally distributed, tests of significance of the regression coefficients were performed using permutations of the residuals of the model.

After applying an exploratory analysis using *p* = 0.05, sufficient power (*β = *0.8) was obtained to detect an effect size as large as *f* = 0.25, corresponding to an explained variance of 6%. A more restrictive value of *p = *0.005 allowed the detection of an effect size as large as *f* = 0.31 with enough power, corresponding to an explained variance of 9%. Even though this level of power seems adequate to identify medium to large [23] differences among groups, the sample size included in this study should be considered as small compared to standard studies. Both the low incidence/prevalence of acromegaly (incidence 3–4 cases per million/year, prevalence 40–60 cases per million) and the strictness of the inclusion criteria precluded us from obtaining a larger sample size. Even though this could be seen as a limitation of the study, it allowed us to perform a very exhaustive clinical assessment of patients (compliance mainly), otherwise unfeasible in larger samples.

GPower 3 served for the analysis of power (http://www.psycho.uni-duesseldorf.de/aap/projects/gpower/), R 2.13.0 (http://www.r-project.org/) and package ‘lmPerm’ (available in the CRAN repository (http://cran.r-project.org/web/packages/lmPerm/) served for all the other statistical analyses.

## Results

### Patient Characteristics

Patients’ clinical characteristics are shown in Table 1. No differences in the age at acromegaly diagnosis between groups were found (*p = *0.748). As expected, cured patients showed normal GH and IGF-I values at assessment. Conversely, all naïve acromegaly patients showed IGF-I levels above the normal age-specific range (>3 SD) and GH hypersecretion (*F* = 17.48, *p*<0.001), as compared to cured patients). We should note that the hormonal analysis showed higher mean PRL serum levels in naïve patients (*F* = 4.79, *p = *0.038), but this was due to the inclusion of patients with mixed GH/PRL secreting adenoma. As average, comorbidities were fewer in cured acromegaly patients as compared to naïve patients (*F* = 11.46, *p = *0.001), as previously reported [1], [24]. Nevertheless, no significant differences in number of patients with hypertension (*p = *0.191), diabetes (*p = *0.661) or hypopituitarism (*p = *0.301) between groups were found. Similarly, no differences in tumor characteristics (size and extension) (all *p-values* >0.647) and mean duration of untreated disease (*p = *0.983) were found between patient groups.

**Table 1:** Characteristics of the patient samples included in the study.

	Cured acromegaly (N = 20)	Naïve acromegaly (N = 20)
Age at diagnosis, mean (SD)	46.40 (11.08)	48.50 (10.25)
GH (µg/liter), mean (SD)	0.40 (0.51)	16.32 (14.72)
IGF-I (µg/liter), mean (SD)	179.63 (104.70)	1022.28 (507.16)
IGF-I_SD_, mean (SD)	0.37 (1.83)	16.29 (8.39)
Prolactin (µg/liter), mean (SD)	7.22 (3.41)	25.03 (30.27)
**Surgery, n (% of patients)**		
Transphenoidal	20 (100)	0
No	0	20 (100)
**Comorbidities, n (% of patients)**		
Myocardiopathy	2 (10)	2 (10)
Hypertension^a^	5 (25)	10 (50)
Arthralgia	2 (10)	2 (10)
Carpal tunnel syndrome	1 (5)	6 (30)
Diabetes mellitus^b^	2 (10)	4 (20)
Sleep apnea	1 (5)	5 (25)
Malignancy	1^c^ (5)	0
Dyslipidemia	3 (15)	4 (20)
Gallstones	1 (5)	4 (20)
Hypopituitarism (any axis)	8 (40)	4 (20)
ACTH	2 (10)	2 (10)
TSH	5 (25)	2 (10)
FSH/LH	5 (25)	1 (5)
Depression	6 (30)	3 (15)
**Tumor characteristics, n (% of patients)**		
Macroadenoma	13 (65)	17 (85)
Microadenoma	7 (35)	3 (15)
Suprasellar extension	4 (20)	4 (20)
Cavernous sinus extension	7 (35)	7 (35)
Sphenoid sinus extension	0	2 (10)
GH secreting adenoma	14 (70)	n/a
Mixed GH/PRL secreting adenoma	6 (30)	n/a
Duration of untreated disease, mean months (SD)	59.65 (55.79)	59.32 (40.88)
Duration of biochemical remission mean months (SD)	95.73 (59.01)	n/a
AcroQoL score^d^	31.85 (25.28)	42.82 (24.40)

a Treatment in the cured acromegaly group (n): angiotensin-converting enzyme inhibitors (4); angiotensin II type 1 receptor blockers (1). Treatment in the naïve acromegaly group: angiotensin-converting enzyme inhibitors (6); calcium channel blocker (3); angiotensin II type 1 receptor blockers (1).

b All the patients were treated with metformin for diabetes. Only one patient (cured acromegaly) required additional insulin treatment.

c Colorectal cancer.

d The higher the score in this questionnaire, the worse self-perceived QoL is.

### Cognitive Function after Cure of Acromegaly

Means and standard deviations of the scores of neuropsychological tests are presented in Table 2. Overall, acromegaly patients displayed poorer working memory performance compared to healthy controls. Main effect of Group was significant in the backward task of the Digit Span Test (*F* = 7.01, *p-perm* = 0.008). This effect remained significant after adjusting by covariates (*p-perm* = 0.046). Post hoc analysis revealed that naïve patients performed worse in the backward task of the Digit Span test than healthy subjects (*p*<0.05, Bonferroni corrected), while naïve and cured patients did not differ significantly in this test (*p = *0.220). Differences in several indexes of the LMW-R that accounts for learning capability and accuracy, such as the True Recall Index (*F* = 3.23, *p = *0.034; cured vs. healthy subjects post hoc comparison, *p*<0.05, Bonferroni corrected) and the Confabulation Index (*F* = 4.37, *p = *0.034, naïve vs. healthy subjects post hoc comparison, *p*<0.01, Bonferroni corrected) were also observed.

**Table 2:** Neuropsychological test results for acromegaly groups and healthy controls.

	Cured patients	Naïve patients	Healthy subjects
**Attention**			
Simple attention test, RT (sec.)	0.488 (0.06)	0.467 (0.06)	0.449 (0.06)
Conditional attention test, RT (sec.)	0.387 (0.13)	0.399 (0.05)	0.403 (0.08)
Trail Making Test A, time (sec.)	56 (30.04)	61.1 (27.92)	39.5 (9.26)
**Executive functioning**			
Trail Making Test B, time (sec.)	135 (77.24)	186.4 (93.48)	89.6 (25.71)
Stroop Color-Word Test, RT (sec.)	1.62 (0.39)	1.64 (0.59)	1.44 (0.31)
Tower of Hanoi test, total time (sec.)	177 (144.32)	246 (177.70)	154 (112.19)
**Memory**			
Digit Span Test (forward)	7.60 (1.95)	6.94 (1.60)	8.17 (1.11)
Digit Span Test (backward)	5.33 (1.50)	4.13 (1.53)a,d	6.17 (1.26)
LMW-R			
True Recall Index	8.29 (1.01)a,e	8.36 (0.83)	8.98 (0.50)
Confabulation Index	4.56 (3.43)	5.93 (3.44)b,e	2.43 (1.57)
Delayed recall	6.94 (1.09)c,f	7.40 (1.45)c,f	9.25 (0.90)
Complex Figure Test			
Copy condition	32.5 (4.31)	32.8 (5.81)	35.2 (1.19)
Memory condition	20.3 (5.78)a,g	15.3 (6.87)c,g	26 (4.22)

Means (SD) are shown.

a *p<*0.05, vs. healthy subjects comparison.

b *p*<0.01, vs. healthy subjects comparison.

c *p*<0.001, vs. healthy subjects comparison.

d Median score in normative data = -1.77.

e These indexes do not have normative data available.

f Median score in normative data: cured patients = −1.68 (35% of patients scored below −1.5 SD); naïve patients = −2.21 (50% of patients scored below −1.5 SD); healthy subjects = 0.14 (no patient scored below −1.5 SD).

g Median percentile with test’s normative data: cured patients = 12.5 (40% of patients below 10th percentile); naïve patients = 4 (70% of patients below 10th percentile); healthy subjects = 50 (no patient below 10th percentile).

Note: LMW-R = Luria’s Memory Words Test-Revised.

Patients also performed worse than healthy controls in tests assessing delayed memory recall. Performance of cured patients was also impaired in these tests, showing levels similar to those observed in naïve patients. More specifically, our analysis revealed that both cured and naïve patients performed worse in the test assessing delayed verbal recall (LMW-R) than healthy subjects (*p*<0.0001 in both comparisons, Bonferroni corrected) while patients’ performance in the immediate recall condition was similar to that of healthy subjects (*p*>0.102). In addition, patients performed worse in the memory condition of the Complex Figure Test (*F* of the interaction = 4.78, *p* = 0.003; post hoc pairwise comparisons: naïve vs. healthy, *p*<0.0001, cured vs. healthy, *p*<0.05, naïve vs. cured, *p*<0.10, Bonferroni corrected), which assesses visual recall while no differences among the three groups were observed in the copy condition.

**Figure 1:**
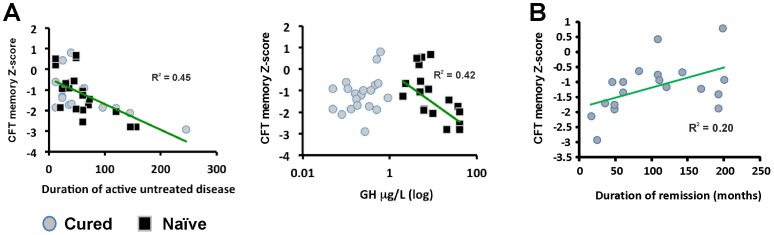
Relationship between clinical predictors obtained from multivariate regression analysis and cognitive data. (**A**) longer duration of untreated acromegaly (in months) is independently associated with worse visual memory performance. Higher GH levels are independently associated with worse visual memory recall in patients with naïve acromegaly, but not in cured patients. (**B**) shorter biochemical remission is positively associated with worse visual memory recall. CFT, Complex Figure Test. Partial *R^2^* were calculated controlling for the other variables in the model.

We performed multiple linear regression analysis, as described above, on delayed memory recall tests (CFT and LMW-R). In CFT, the regression analysis (adjusted R^2^ = 0.315, *p = *0. 0.007) revealed that duration of untreated acromegaly was independently associated with poor memory performance in both naïve and cured patients (*β* = −1.095, *t* = −2.082, *p-*perm = 0.004). Acromegaly patients (both naïve and cured) with longer exposure to untreated GH/IGF-I excess performed worse in the CFT delayed recall test. The naïve group also displayed a significant negative relationship between GH levels and performance in CFT delayed recall (*β* = −1.657, *t* = −2.565, *p-*perm = 0.011) (Fig. 1A). However, a trend towards worse visual memory performance in both naïve and cured patients was observed when hypopituitarism entered the analysis (*β* = −0.066, *t* = −1.861, *p-*perm = 0.074), indicating that hypopituitarism was associated with lower scores in the CFT test. Furthermore, duration of biochemical remission was positively associated with performance on the CFT test (*p-*perm = 0.021) (Fig. 1B). The regression analysis did not provide a reliable predictor for the LMW-R test (delayed verbal recall) (*p = *0.151; data not shown).

### Neurophysiological Function after Cure of Acromegaly

**Figure 2:**
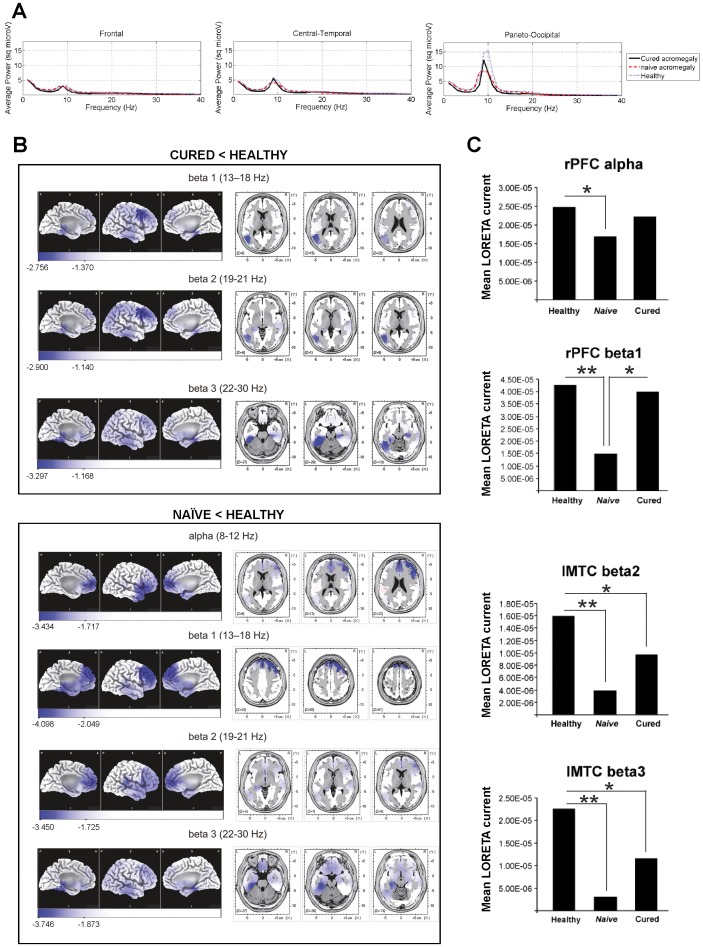
Neurophysiological results of patients with acromegaly. (**A**) Power spectra differences (frequency range: 1–40 Hz) among patient groups and healthy controls. Decreased EEG power was observed in fast bands (alpha and beta) for both patient groups. Note the decreased power in patients with acromegaly at peak alpha frequency, 10 Hz. (**B**) LORETA comparisons between acromegaly patients and healthy controls. Upper panel: comparison between cured patients and healthy subjects. Highest significant LORETA differences were found in left medial temporal cortex in beta 2 band (4 significant neighboring voxels, 1.37 cm^3^, *p*<0.04) and beta 3 band (15 significant neighboring voxels, 5.14 cm^3^, *p*<0.007). Cured patients showed significantly lower LORETA activity than healthy subjects in cortical areas shown in blue. Lower panel: comparison between naïve patients and healthy subjects. Highest significant LORETA differences were found in left medial temporal cortex in beta 2 band (8 significant neighboring voxels, 2.74 cm^3^, *p*<0.012) and beta 3 band (30 significant neighboring voxels, 10.29 cm^3^, *p*<0.005) and in right dorsolateral PFC in alpha (8 significant neighboring voxels, 2.74 cm^3^, *p*<0.012) and beta 1 (28 significant neighboring voxels, 9.604 cm^3^, *p*<0.006) bands. Naïve patients showed significantly lower LORETA activity than healthy subjects in cortical areas shown in blue. Scales show maximal *t* value and maximal significant *t* value (*p*<0.05, corrected for multiple comparisons). (**C**) ROI analysis comparisons among groups. ROI LORETA activity in acromegaly patients is reduced as compared to healthy subjects. *p<0.05; **p<0.01, corrected for multiple comparisons.

Significant differences in EEG power spectra recorded at parieto-occipital locations were found between the three groups. More specifically, differences in parieto-occipital alpha (*F* = 7.487, *p*<0.0001), beta 2 (*F* = 4.415, *p* = 0.015), beta 3 (*F* = 3.805, *p* = 0.028), and gamma (*F* = 3.102, *p* = 0.015) were found between the three groups. Post hoc comparisons revealed decreased alpha and beta 2 power (sqmicroV) in both cured and naïve patients compared to healthy subjects (all *p*<.05). Interestingly, no significant differences were found in faster frequency bands between cured patients and healthy subjects, whereas naïve patients displayed significant power decrease in beta 3 (*p*<0.02) and gamma (*p = *0.045) bands compared to healthy controls (Fig. 2A).

LORETA analysis of the whole solution space identified two cortical areas with the highest reliable effects. Maximal statistical differences were found in the right dorsolateral PFC, in the 22–40 Hz range (*F* = 3.785, *p* = 0.012), and in the left parahippocampal cortex, in the 8–18 Hz range (*F* = 4.604, *p = *0.006) (Fig. 2B). ROI analysis (Fig. 2C) supported these findings, with naïve and cured patients displaying decreased beta 2 and 3 activity in the left MTC. In addition, decreased alpha activity in the right PFC was found in naïve patients compared to healthy subjects. LORETA source analysis for beta 1 also showed decreased right PFC activity in naïve patients compared to cured patients.

In terms of Cohen [23], weak to moderate correlations were found between LORETA ROI activity and memory performance, particularly between CFT performance and left MTC beta 3 activity, for both patient groups. A significant correlation was also found between CFT and right PFC beta 1 activity in the naïve group (Table 3).

**Table 3:** Correlation coefficients (Pearson’s *r*), with associated *p* values (calculated after 5,000 randomizations) for the correlation analysis between LORETA ROI activity and performance in delayed recall tests.

	Cured (N = 20)		Naïve (N = 20)		Overall	
	LMW-R	CFT	LMW-R	CFT	LMW-R	CFT
rPFC alpha	–.33(.140)	−.02 (.940)	.39 (.095)	.36 (.124)	.19 (.240)	.11 (.497)
rPFC beta 1	−.34 (.139)	.36 (.129)	.23 (.351)	**.67 (.002)**	−.01 (.928)	.27 (.102)
lMTC beta 2	.26 (.275)	.43 (.059)	.09 (.696)	.23 (.351)	.11 (.497)	.24 (.147)
lMTC beta 3	.33 (.160)	**.62 (.004)**	.05 (.984)	**.69 (.001)**	.05 (.746)	**.63 (<.001)**

Significant associations shown in bold.

Note: LMW-R = Luria’s Memory Words Test-Revised; CFT = Complex Figure Test.

**Figure 3:**
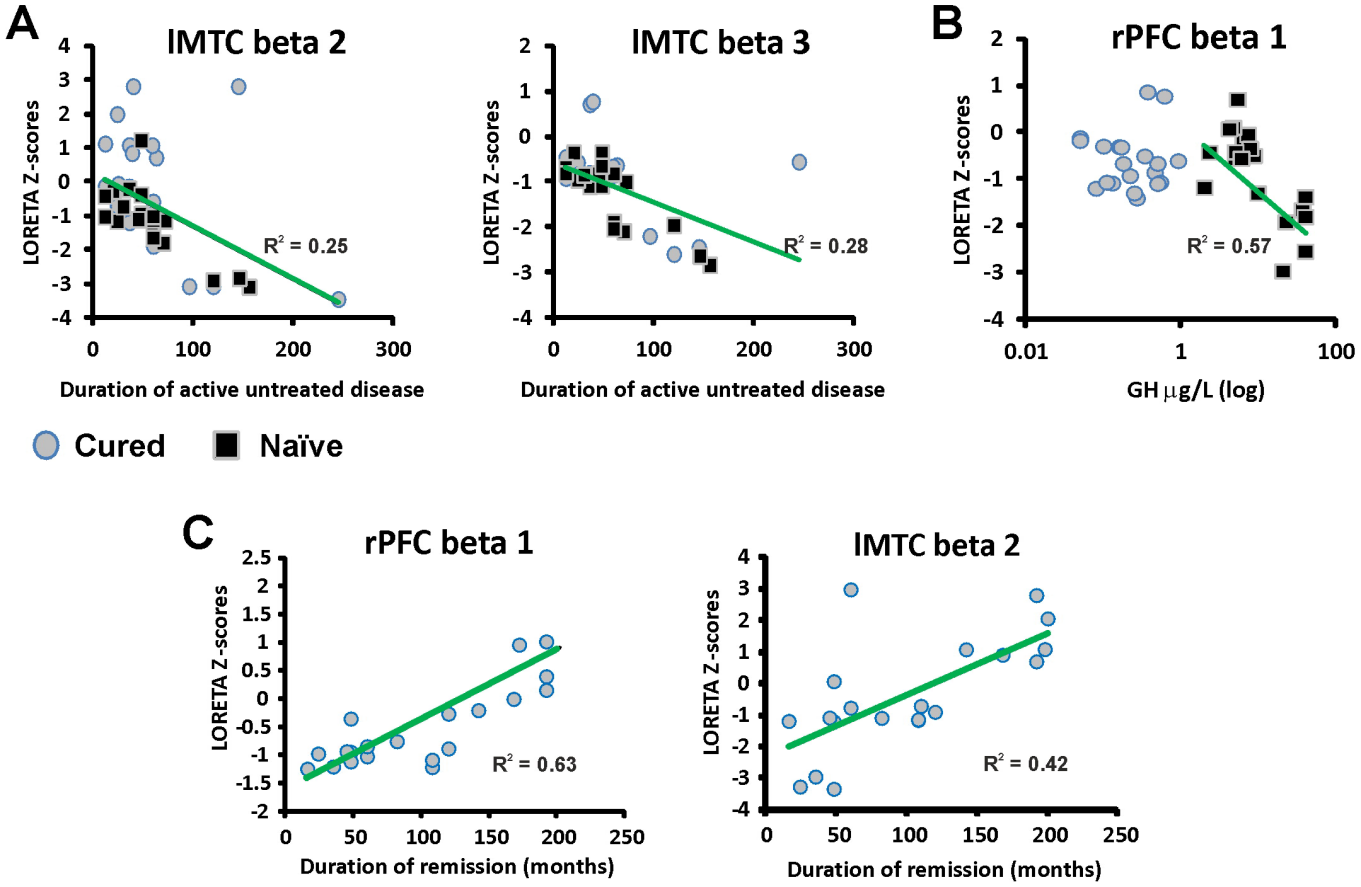
Relationship between clinical predictors obtained from multivariate regression analysis and LORETA data. (**A**) lower LORETA Z-scores in left medial temporal cortex were associated with longer duration of untreated acromegaly. (**B**) higher GH levels were associated with decreased LORETA Z-scores in rPFC in patients with naïve acromegaly, but not in cured patients. (**C**) lower LORETA Z-scores in beta lMTC and rPFC was associated to shorter biochemical remission. rPFC, right prefrontal cortex; lMTC, left medial temporal cortex. Partial *R^2^* were calculated controlling for the other variables in the model.

Multiple regression analysis of LORETA activity (beta 2: adjusted R^2^ = 0.313, *p*<0.0001; beta 3: adjusted R^2^ = 0.494, *p*<0.0001) revealed significant negative relationships between LORETA beta 2 (*β* = −0.009, *t* = −2.039, *p-*perm = 0.049) and beta 3 (*β* = −0.014, *t* = −2.710, *p-*perm = 0.014) activities in the left MTC and the duration of untreated disease, for both cured and naïve groups. In other words, longer exposure to high levels of GH and IGF-I was associated with weaker LORETA activity in left middle temporal areas (Fig. 3A).

Neither age at diagnosis, hypertension or diabetes was associated with MTC LORETA activity (*p-*perm>0.12). Patients with hypopituitarism, regardless of the patient group, showed a trend of having less LORETA activity in left MTC beta 2 and beta 3 (*p-*perm = 0.060 and 0.051, respectively). We also observed a negative relationship between right PFC LORETA beta 1 activity and GH levels in patients with naïve acromegaly (*β* = −0.156, *t* = −3.428, *p-*perm = 0.002), but not in cured patients (Fig. 3B). In addition, duration of biochemical remission correlated positively with right PFC beta 1 (*p-*perm <0.0001) and left MTC beta 2 activities (*p-*perm = 0.001). Duration of remission was not significantly associated with any other ROI’s activity (*p-*perm >0.07).

## Discussion

Contrary to our expectations, cured acromegaly patients displayed specific memory deficits. Thus, our cognitive assessments do not indicate full recovery from neurocognitive impairment after cure of acromegaly, at least in patients with short-term biochemical remission.

Accumulating evidence indicates that neurocognitive impairment should be added to the list of complications associated with acromegaly. Sievers et al. [5] reported that almost 70% of acromegaly patients of their cohort performed below normal levels in at least one cognitive test (memory, attention or executive functions). Our results showed a lower prevalence of cognitive deficits in acromegaly patients, but it is important to note that the Sievers study included a heterogeneous sample group comprised of patients with a history of radiotherapy (25%) or uncontrolled disease (40%), which may explain the discrepancy between these results. Our study also shows that patients with surgically cured acromegaly display significant power and topographical EEG alterations similar to those registered in naïve patients. Using EEG power spectra and intracortical tomography (LORETA), we found that cured patients showed lower alpha and beta cortical activity in left medial temporal cortex than the healthy group. These differences in the LORETA analysis correlated positively with the performance on the memory condition of the CFT, which assesses visuospatial memory. The delayed recall condition of the CFT is perhaps the most widely used neuropsychological test in clinical settings to study the non-verbal aspect of memory [25]. This test has been classically used to assess memory functions that rely on structures located in the right hemisphere, especially hippocampus and associated regions in the medial temporal lobe [26], [27]. In our study, our analysis revealed a significant association between performance on this test and left MTC activity suggesting that this area might have an important role on non-verbal memory processes. In agreement with this hypothesis, neuropsychological studies have found that patients with left hemisphere stroke also show impairment in this test [28], whereas other studies have not found an association between performance on CFT and right temporal lobe lesions [29]. A potential explanation for these findings is that verbal encoding (which relies more on the left hemisphere) might be necessary for the patient to storage a large number of different geometrical figures and their positions [30].

Our analysis expands and refines previous results (including our own) by identifying a number of variables that independently predict the presence of neurocognitive impairment in acromegaly, even after cure. Lower memory scores in both patient groups were associated with longer duration of untreated disease (i.e., longer exposure to high levels of GH and IGF-I). The fact that prolonged exposure was related to neurocognitive outcome in both cured and naïve acromegaly is not entirely surprising. Longer exposure to GH/IGF-I hypersecretion has been linked to an increased prevalence of other systemic complications, namely cardiovascular or joint disorder, which often persist after cure of the disease [1], [24]. Interestingly, previous studies have linked the duration of untreated active disease to volumetric changes in global brain volume as well as left and right hippocampus in acromegaly patients [5], [7].

The regression analysis also revealed that GH levels predicted PFC beta activity in naïve acromegaly but not in cured patients. Noteworthy, this area showed decreased LORETA activity as compared to both cured and healthy subjects. These observations suggest that neurocognitive deficits are linked to hypersecreted GH levels, thus providing a possible explanation for the slightly better neurocognitive state (especially in learning processes) of cured patients observed in our study. This is also supported by the fact that a significant association between right PFC beta activity and CFT performance was found only in naïve patients. This data suggests that hypoactivity in PFC might contribute to memory deficits in naïve acromegaly patients.

Based on previous results [5], we expected that patients with acromegaly would show deficits in executive functions, especially in those patients with decreased PFC LORETA activity. However, we could not detect statistically significant differences among the different groups. This could be due to the sample size of our study that was not adequate to detect subtle differences (due to the strict exclusion criteria used) in cognitive functions linked to the PFC. This is also supported by the power analysis we performed, which indicated that only moderate to large effects could be detected. Additional studies with larger sample sizes are necessary to conclusively demonstrate whether non-memory related cognitive domains are affected in acromegaly patients.

Another important result from our study is that longer biochemical remission of acromegaly is associated to better neurocognitive status. This result raises the question whether neurocognitive impairment might improve after long-term remission. In agreement with this hypothesis, another study reported low prevalence of memory deficits in a cohort of acromegaly patients with longer duration of remission [6] (mean duration of remission of 13.1±1.0 years compared to 8.35±1.17 years in our study). Likewise, a recent study on patients with median biochemical remission of 8 years showed similar results to ours [31]. Prospective studies with longer follow up are needed to conclusively prove full recovery from neurocognitive impairment in cured acromegaly patients.

The mechanisms by which increased GH levels might alter brain functions remain purely speculative but it is important to note that GH and IGF-I can cross the brain-blood barrier [25] and thus, these factors might directly affect brain structures relevant to cognitive functions. In this regard, increased hippocampal volume has been described in acromegaly patients although it is unclear whether this is due to increased neurogenesis in hippocampus [5], [7]. Interestingly, in animal studies increased GH/IGF-I signaling promotes neurogenesis in the dentate gyrus [32]–[34]. However, other studies have shown that brain overgrowth mediated by GH/IGF-I hypersecretion is due to increased gliogenesis and glial activity (increased myelin production) rather than increased neurogenesis [35], [36]. Further studies are required to determine the neurobiological basis of the alterations in brain structure and function observed in acromegaly patients. Interestingly, both decreased and excess GH and IGF-I levels have been linked to alterations in cognitive functions. Falleti et al. [37] reported moderate impairment in memory and executive functions in patients with adult-onset GH deficiency. However, whether excessive and defective GH secretion cause these deficits through common mechanisms remains to be determined. Whatsoever the pathophysiological commonalities between GH/IGF-I excess and deficiency might be, cumulative data indicate that GH/IGF-I circulating levels must be kept within a very specific range for proper brain function.

We acknowledge that our data suggests but does not prove a causal relationship between GH/IGF-I overproduction and neurocognitive impairment in acromegaly. As in other chronic diseases, patients with acromegaly may suffer from neurocognitive disturbances as a consequence of more general factors (e.g. decreased QoL or psychiatric disorders). Other cross-sectional studies have tried to address this issue by including patients with non-functioning pituitary adenoma (NFPA) as a comparison group, but controversial results have been obtained [6], [31]. Specific comorbidities associated to acromegaly might also potentially have an impact in the neurocognitive impairment observed in our study. For example, hypertension, diabetes and hypopituitarism may, *per se*, alter brain structure and function. Our study found that these variables had little impact on neurocognitive functions. Unfortunately, MRI/CT scans of brain parenchyma were not available in most of the patients in our study. However, previous work using magnetic resonance imaging failed to find a relationship between white matter lesions (likely due to vascular risk factors) and cognitive impairment, suggesting that cognitive impairment is not mainly due to neurovascular pathology in acromegaly [5].

In conclusion, our study reveals that patients after cure of acromegaly show specific memory deficits and EEG alterations. Our results indicate that treatment outcome (surgically cured acromegaly) did not include full recovery from neurocognitive complications. Neurocognitive complications after post-operative biochemical remission of acromegaly are related to prolonged GH/IGF-I hypersecretion before surgery, although the underlying mechanisms remain unknown. Finally, our data show that patients with longer duration of remission display better neurocognitive functions. In the realm of clinical endocrinology, these results should be considered for the long-term prognosis of neurocognitive complications of patients with acromegaly.

## Supporting Information

Figure S1 **Flow chart of patients who met inclusion/exclusion criteria for the study population.**
(DOC)

Table S1 **Basic demographics of the three groups.**
(DOC)

File S1 **Supporting methods.**
(DOCX)
