# Supplementary material for: Neurocognitive Function in Acromegaly after Surgical Resection of GH-Secreting Adenoma versus Naïve Acromegaly
Source: PLoS One. 2013 Apr 4;8(4):e60041. doi: 10.1371/journal.pone.0060041 (PMC3617159; doi:10.1371/journal.pone.0060041)
Supplement: Figure S1 — Flow chart of patients who met inclusion/exclusion criteria for the study population. (DOC) [file pone.0060041.s001.doc]

**Figure S1. Flow chart of patients who met inclusion/exclusion criteria for the study population**


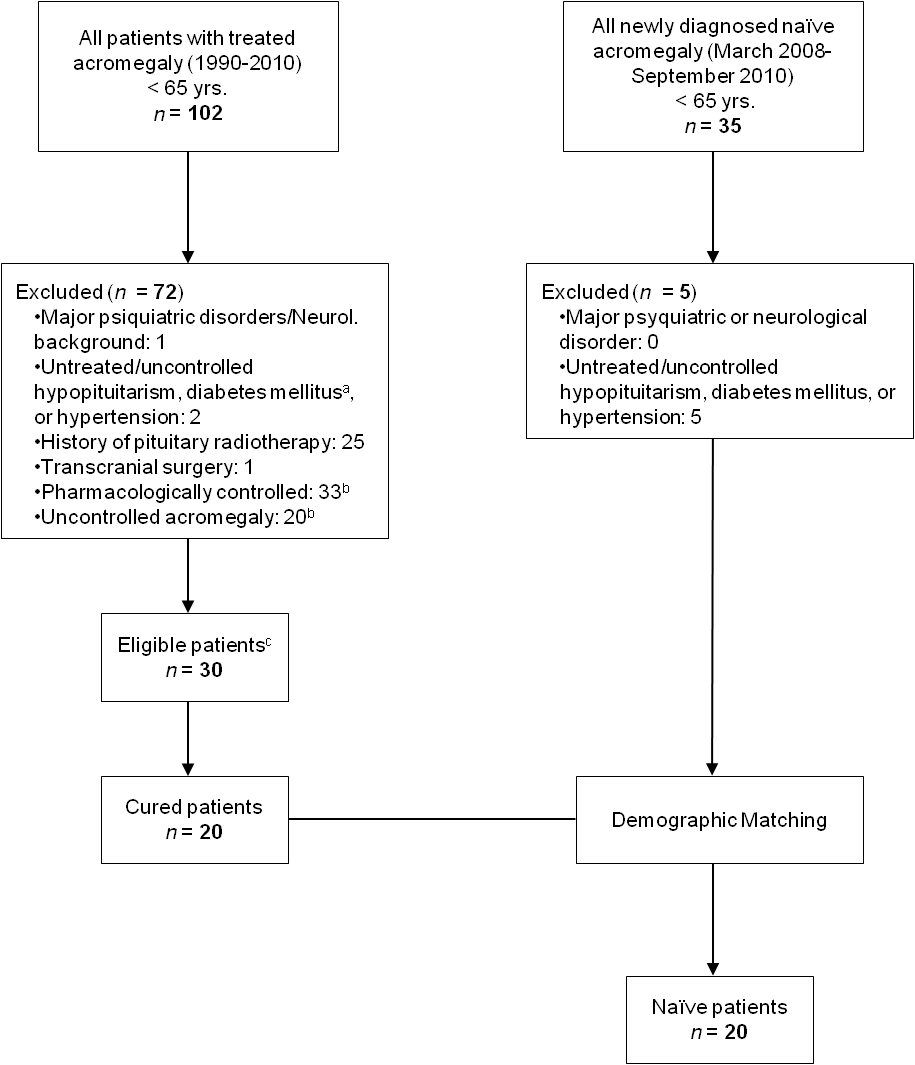


^a^ Uncontrolled diabetes was defined as a fasting plasma glucose >140 mg/dl and HbA1c >7.0% in treated patients.

^b^ Controlled and uncontrolled acromegaly according to the latest consensus acromegaly ‘control’ criteria [13].

^c^ Eight patients declined to participate. Two patients did not complete the assessment.
