# Supplementary material for: Neurocognitive Function in Acromegaly after Surgical Resection of GH-Secreting Adenoma versus Naïve Acromegaly
Source: PLoS One. 2013 Apr 4;8(4):e60041. doi: 10.1371/journal.pone.0060041 (PMC3617159; doi:10.1371/journal.pone.0060041)
Supplement: Table S1 — Basic demographics of the three groups. (DOC) [file pone.0060041.s002.doc]

**Table S1.** **Basic demographics of the three groups.**

|  | Cured acromegaly | Naive acromegaly | Healthy controls | P value |
| --- | --- | --- | --- | --- |
| Age at testing, mean (SD) | 50.60 (10.97) | 49.06 (11.58) | 45.83 (12.45) | 0.447^a^ |
| Sex (% women) | 75 | 75 | 70 | 1^b^ |
| Education (%) | primary: 60  secondary: 20  university: 20 | primary: 60  secondary: 20  university: 20 | primary: 50  secondary: 30  university: 20 | 1^b^ |

a. One-way ANOVA

b. Fisher's exact test approximated using a Monte-Carlo approach
