# Supplementary material for: Neurocognitive Function in Acromegaly after Surgical Resection of GH-Secreting Adenoma versus Naïve Acromegaly
Source: PLoS One. 2013 Apr 4;8(4):e60041. doi: 10.1371/journal.pone.0060041 (PMC3617159; doi:10.1371/journal.pone.0060041)
Supplement: File S1 — Supporting methods. (DOCX) [file pone.0060041.s003.docx]

**SUPPORTING METHODS**

*Hormone evaluation and assays*

Blood samples were collected after overnight fasting. Serum GH levels were measured with a sensitive immunofluorometric assay (Wallac, Turku, Finland), specific for the 22 kDA GH protein, calibrated against World Health Organization International Reference Preparation 80/505 [detection limit 0.01 µg/liter]. Intraassay coefficients of variation (CVs) for GH levels ranging from 2.6–17ng/ml were 5.0 and 5.6%; interassay CVs for these levels were 5.0 and 8.5%. From 2010 on, serum GH concentrations were measured using a solid phase, two-site chemiluminescentimmunometric assay (Siemens, Los Angeles CA, USA), specific for the 22 kDA GH protein, calibrated against WHO NIBSC 98/574 for rGH [detection limit 0.01]. Intraassay CVs for GH levels from 2.6–17 ng/ml were 3.5 and 6.5%; interassay CVs for these levels were 6.5 and 6.6%.

Serum IGF-I levels were measured by a commercial immunoradiometric assay kit after acid ethanol extraction (Immunotech SA, Marseilles, France), with interassay CVs of 3.5% (IGF-I level at 77 ng/ml) and 6.8% (IGF-I level at 1358 ng/ml). Intraassay CVs were 5.0 and 8.0%, respectively. From 2010 onward, serum IGF-1 concentrations were measured using a solid phase, enzyme-labeled immunometric assay (Siemens, Los Angeles CA, USA). Intraassay CVs were 5.0 and 7.5% at mean plasma levels of 8 and 75 nmol/liter, respectively. IGF-I levels were expressed as SD score, using log-transformed IGF-I measurements in 405 healthy individuals [1].

Serum prolactin was measured by an electrochemiluminiscense immunoassay (Modular Analytic E170; Roche Diagnostic, Switzerland), with 0.8% and 1.7% within-run precision and 1.4%–2% total precision (PRL levels from 7.6–210 ng/ml).

Serum free testosterone levels were measured by radioimmunoassay (RIA) methods (FTestosterone RIACT kit, BIOCODE, Liege, Belgium), with 0.1 pg/ml sensitivity. Interassay and intraassay CVs were 5.3% and 3.8%, respectively (normal range: age < 40, range 62.2–142.2 nmol/ l; age 40–49, range 55.5–114.5 nmol/ l; age 50–59, range 45.2–107.6 nmol/ l; age 60–69, range 39.1–90.2 nmol/ l; age > 70, range 31.2–86.7 nmol/l, respectively). Male hypogonadism (n = 2) was treated with testosterone enanthate, 250 mg every 2-3 wk.

Serum estradiol was measured by an electrochemiluminiscense immunoassay (Modular Analytic E170; Roche Diagnostic, Switzerland), with 1.4% to 3.3% within-run precision and 2.2% –4.7%total precision (estradiol levels from 120–13633 pmol/L).

Serum LH was measured by an electrochemiluminiscense immunoassay (Modular Analytic E170; Roche Diagnostic, Switzerland), with 0.6%–1.2% within-run precision and 1.6%–2.2% total precision (LH levels from 5.81–164 IU/L).

Serum FSH was measured by an electrochemiluminiscense immunoassay (Modular Analytic E170; Roche Diagnostic, Switzerland), with 1.3%–2.8% within-run precision and 3.6%–4.5% total precision (FSH levels from 5.30–229 IU/L). Female hypogonadism (n=3) was treated with estradiol patches (25-50 μg, 2 per wk) and progesterone (100 mg for 2 wks).

Serum Cortisol was measured by an electrochemiluminiscense immunoassay (Modular Analytic E170; Roche Diagnostic, Switzerland) with 1.1%–1.7% within-run precision and 1.4%–2.8% total precision (cortisol levels from 4.69–31.40 microg/liter). Serum ACTH levels were measured by chemiluminecentimmunometricassay (Immulite 2000, Siemens, Germany) with intraassay CVs of 3.1%–9.6% and interassay CVs of 5.1%–9.2% (ACTH levels from 2.3–1121 pg/mL). Patients with ACTH deficiency (n=2) were treated with hydrocortisone (15-30 mg/day).

Serum FT4 was measured by aelectrochemiluminiscense immunoassay (Modular Analytic E170; Roche Diagnostic, Switzerland) with 1.4%–2% within-run precision and 2.6%–4.8% total precision (FT4 levels from 9.1–42.7 pmol/L).

Serum TSH was measured by an electrochemiluminiscense immunoassay (Modular Analytic E170; Roche Diagnostic, Switzerland) with 1.2%–3% within-run precision and 3.2%– 7.2% total precision (TSH levels from 0.003–9.37 μIU/mL). Patients with TSH deficiency (n=5) were treated with L-thyroxine (75-150 μg/day).

*Cognitive tests*

Cognitive testing was performed in patients under fed conditions (after breakfast). The neurocognitive assessment was conducted between 10:00 am and 12:00 am, after the neurophysiological exam. The assessment usually lasted 60 minutes, with a 5-minute break halfway through the assessment. Tasks and tests were administered randomly across subjects.

A functional classification was used to subdivide the tests into major cognitive domains. Tests were classified into attention, executive functioning, working memory and delayed verbal and non-verbal memory recall [2]. Basic attention processes, namely vigilance, sustained attention and reaction times, were assessed using a computerized version of the letter cancellation task, included in the Seville Neuropsychological Battery [3]. This test was administered in two different ways: a Simple Letter Cancellation task, in which letters are presented sequentially at a rate of 1/sec and the subject has to detect when the letter 'O' appears; and a Conditional Letter Cancellation task, that is administered in the same format than the Simple version but the subject has to detect when the letter 'O' followed the 'X'. Correctly identified targets and mean reaction times were collected.

Executive functions were assessed with a battery of neuropsychological tests. The Trail Making Test (part B) was used to measure cognitive flexibility and visuo-conceptual tracking. The time used to complete this test as well as the number of errors were measured. The Stroop Color-Word Test was used to assess inhibitory function. The reaction time and the number of correct and wrong responses were counted. The Tower of Hanoi test, a complex problem-solving task, was used to assess planning, goal-directed behavior, and error learning. Results collected for this test included the number of correct movements and errors as well as the total time employed to solve the task.

Memory was assessed using a battery of tests that measured working memory and long-term memory (verbal and visual) and learning processes. Working memory was assessed using the forward and backward versions of the Digit Span Test, included in the Wechsler Memory Scale [4]. The verbal memory was assessed using the Luria's Memory Words Test-Revised (LMW-R) with its learning and delayed recall conditions. The LMW-R is a standard word-list learning test. Briefly, the subject is asked to learn a list of 10 unrelated words over ten consecutive trials. After administering the test, the correct words, repetitions and additions are counted. True Recall Index and Contamination Index are calculated from these variables, which were used to score the learning condition of the LMW-R (Description and calculation of memory indexes provided by LMW-R could be obtained at reference [5]). After a 30-minute delay, subjects were asked to recall the words. The correct number of recalls is used as the score for the delayed recall condition.

Non-verbal memory was assessed with the delayed recall condition of the Complex Figure Test (free recall of the complex figure after a 30 minute delay of the copy condition). Total correct recalls of this condition was used to assess visuospatial memory within declarative memory. Standard administration and correction of this test was performed as previously described [6].

*References*

1. Granada ML, Ulied A, Casanueva FF, Pico A, Lucas T, et al. (2008) Serum IGF-I measured by four different immunoassays in patients with adult GH deficiency or acromegaly and in a control population. Clin Endocrinol (Oxf) 68: 942-950.
2. Lezak M (1995) Neuropsychological assessment. New York: Oxford University Press.
3. León-Carrión J (2011). The Seville Neuropsychological Test Battery (BNS). Available: http://www.neurobirds.com/. Accessed 28 October 2012.
4. Weschler D (2000) Wechsler Memory Scale – Third Edition (WMS-III). Madrid: TEA Ediciones.
5. León-Carrión J, Atutxa AM, Mangas MA, Soto-Moreno A, Pumar, A, et al. (2009) A clinical profile of memory impairment in humans due to endogenous glucocorticoid excess. Clin Endocrinol (Oxf) 70: 192-200.
6. Shin MS, Park SY, Park SR, Seol SH, Kwon JS (2006) Clinical and empirical applications of the Rey-Osterrieth Complex Figure Test. Nat Protoc 1: 892-899.
